# Mannose-Functionalized Isoniazid-Loaded Nanostructured Lipid Carriers for Pulmonary Delivery: In Vitro Prospects and In Vivo Therapeutic Efficacy Assessment

**DOI:** 10.3390/ph16081108

**Published:** 2023-08-04

**Authors:** Shaveta Ahalwat, Dinesh Chandra Bhatt, Surbhi Rohilla, Vikas Jogpal, Kirti Sharma, Tarun Virmani, Girish Kumar, Abdulsalam Alhalmi, Ali S. Alqahtani, Omar M. Noman, Marwan Almoiliqy

**Affiliations:** 1 School of Medical and Allied Sciences, G. D. Goenka University, Gurugram 122103, India; vikas.jogpal@gdgu.org (V.J.); kirti.sharma@gdgu.org (K.S.); 2 Department of Pharmaceutical Sciences, Guru Jambheshwar University of Science and Technology, Hisar 125001, India; surbhiraman.rohilla22@gmail.com; 3 School of Pharmaceutical Sciences, MVN University, Palwal 121105, India; tarun.virmani@mvn.edu.in (T.V.); girish.kumar@mvn.edu.in (G.K.); 4 Department of Pharmaceutics, School of Pharmaceutical Education and Research, Jamia Hamdard, New Delhi 110062, India; asalamahmed5@gmail.com; 5 Department of Pharmacognosy, College of Pharmacy, King Saud University, P.O. Box 2457, Riyadh 11451, Saudi Arabia; alalqahtani@ksu.edu.sa (A.S.A.); onoman@ksu.edu.sa (O.M.N.); 6 Department of Translational Molecular Pathology, The University of Texas MD Anderson Cancer Center, Houston, TX 77030, USA; msmohammed@mdanderson.org

**Keywords:** isoniazid, nanostructured lipid carriers, in vivo pharmacokinetics, drug release profile, histopathological toxicity, mannosylation

## Abstract

Resistance to isoniazid (INH) is common and increases the possibility of acquiring multidrug-resistant tuberculosis. For this study, isoniazid-loaded nanostructured lipid carriers (INH-NLCs) were developed and effectively functionalized with mannose (Man) to enhance the residence time of the drug within the lungs via specific delivery and increase the therapeutic efficacy of the formulation. The mannose-functionalized isoniazid-loaded nanostructured lipid carrier (Man-INH-NLC) formulation was evaluated with respect to various formulation parameters, namely, encapsulation efficiency (EE), drug loading (DL), average particle size (PS), zeta potential (ZP), polydispersity index (PDI), in vitro drug release (DR), and release kinetics. The in vitro inhalation behavior of the developed formulation after nebulization was investigated using an Andersen cascade impactor via the estimation of the mass median aerosolized diameter (MMAD) and geometric aerodynamic diameter (GAD) and subsequently found to be suitable for effective lung delivery. An in vivo pharmacokinetic study was carried out in a guinea pig animal model, and it was demonstrated that Man-INH-NLC has a longer residence time in the lungs with improved pharmacokinetics when compared with unfunctionalized INH-NLC, indicating the enhanced therapeutic efficacy of the Man-INH-NLC formulation. Histopathological analysis led us to determine that the extent of tissue damage was more severe in the case of the pure drug solution of isoniazid compared to the Man-INH-NLC formulation after nebulization. Thus, the nebulization of Man-INH-NLC was found to be safe, forming a sound basis for enhancing the therapeutic efficacy of the drug for improved management in the treatment of pulmonary tuberculosis.

## 1. Introduction

Tuberculosis is widely spread pandemic disease caused by the bacteria *Mycobacterium tuberculosis*, and it remains a common cause of death despite the availability of effective treatment [1,2]. The major drawback of the current drug treatments is the emergence of resistance, i.e., extensive drug-resistant (XDR) tuberculosis and multidrug-resistant (MDR) tuberculosis [3]. This occurs due to high doses and long-term treatment plans that trigger the natural process of spontaneous chromosomal mutations in mycobacteria linked to inadequate treatment outcomes, post-treatment relapse, and death [4,5]. To eradicate this life-threatening disease, drug doses and dosing frequencies must be considerably reduced to reduce resistance and increase the effectiveness of treatments [6,7].

Isoniazid is the safest first-line antitubercular drug, and it acts by suppressing mycolic acid synthesis (a basic unit of the bacterial cell wall) [8]. It comes under BCS class III (high solubility and limited permeability). It is rapidly absorbed and achieves peak plasma concentration within 1–3 h post-oral administration [9]. It has a very short half-life of 1–2 h and requires frequent dosing. The recommended dose for isoniazid ranges from 5 to 10 mg/kg/day. Although, a higher dose (up to 25 mg/kg/day) can also be prescribed in severe cases [10]. The bacteria quickly become resistant to INH due to patient non-adherence, which has hampered the effectiveness of the drug [11]. So, there is a need to redevelop the conventional formulations of isoniazid with an emphasis on targeting efficiency, decreasing dosage and dosing frequency, and increasing the therapeutic efficacy of treatment [12].

Passive and active targeting approaches are widely used to achieve targeted therapeutic delivery to the lungs for the successful treatment of tuberculosis [13]. Passive targeting can be achieved by maintaining an optimal mean particle diameter of 200 to 600 nm to deposit the particles deep into the lungs [14,15]. Active targeting can be achieved via mannose functionalization of the formulation as it is more capable of delivering a high drug payload to the target site and increasing the mean residence time in the lungs compared to conventional dosage forms [16,17,18,19,20,21].

Nanostructured lipid carriers have been exploited as drug delivery carriers and are enhanced versions of the solid lipid nanoparticles employed in the development of lipid nanocarrier formulations [22,23]. The inclusion of liquid lipids in the formation of nanostructured lipid carriers is crucial as it significantly enhances the properties of the formulation [24]. NLCs are advantageous as they are capable of overcoming the limitations of solid lipid nanoparticles, such as the poor drug loading of hydrophilic drugs, low physical stability, and degradation of the loaded bio-actives, and provide high drug loading capacity [25,26]. NLCs can incorporate both hydrophilic and lipophilic drugs, providing a sustained release effect with high in vivo tolerance and facilitating administration via various routes [27].

This work aimed to formulate isoniazid-loaded nanostructured lipid carriers using solid lipids, such as compritol 888 (COMP) and octadecyl amine (ODA); a liquid lipid, namely, linoleic acid (LA); and surfactants, namely, tween 80 and poloxamer 40. This formulation was functionalized using D-mannose to enhance the specificity and delivery of the nanocarrier formulation deep into the lungs. The developed Man-INH-NLC formulation was characterized for encapsulation efficiency, drug loading, average particle size, polydispersity index, and zeta potential. In vitro drug release was estimated for both INH-NLC and Man-INH-NLC in a suitable medium at different pH to simulate the different parts of the lungs. Surface morphology was observed via TEM photomicrographs. The in vitro behavior of the nebulized mist was also investigated by estimating the mass median aerosolized diameter and geometric aerodynamic diameter, output efficiency (OE), and respirable fraction (RF). Furthermore, an in vivo pharmacokinetic and bioavailability assessment was carried out using a guinea pig animal model. Histopathological studies were also conducted, and hepatotoxicity was investigated by assessing the toxicity of the formulation.

## 2. Results and Discussion

### 2.1. Investigation of Mannose-Functionalized NLC

**Figure 1:**
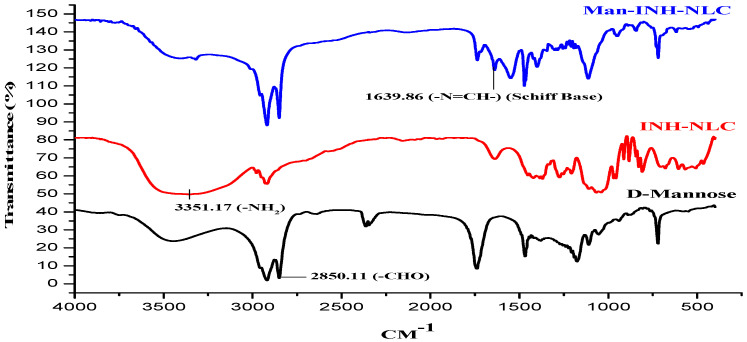
FT-IR spectra of INH–NLC and Man–INH–NLC for the detection of Schiff base (–N=CH–).

The INH-NLC formulation was functionalized with mannose for specific delivery into the lungs. For this purpose, the mannose ring opening phenomenon was used. The acidic environment provided by the acetate buffer results in the opening of the mannose ring. A Schiff’s base (–N=CH–) was formed due to the interaction between the aldehydic group of D-mannose and the octadecylamine amino group of the INH-NLC formulation. Figure 1 shows a characteristic absorption band of the aldehydic -C(H)=O mannose group at around 2850.11 cm^−1^, whereas the absorption band at 3351.17 cm^−1^ represents the -NH_2_ group of octadecylamine in the spectrum of INH-NLC formulation. An interaction between these two groups resulted in the constitution of Schiff’s base, which was confirmed by an absorption band at around 1639.86 cm^−1^ in the FT-IR spectra of the Man-INH-NLC formulation. These observations were similar to that reported in the research work of Pinheiro M. et al., 2016 [28].

### 2.2. Encapsulation Efficiency and Drug Loading

The INH-NLC and Man-INH-NLC formulations were analyzed for EE and DL and found to be decreased insignificantly (*p* > 0.05) for Man-INH-NLC compared to INH-NLC (Table 1). This might be due to the diffusion of some surface-adsorbed drugs in a buffer medium during the process of mannosylation. This finding is consistent with earlier research [29].

### 2.3. Average Particle Size, Polydispersity Index, and Zeta Potential Measurements

The average particle size of INH-NLC and Man-INH-NLC formulations are presented in Table 2. The Mannosylation process resulted in a substantial increase in particle size due to mannose functionalization on the surface of INH-NL [30]. For effective passive targeting, the particle size should be in the range of 200 to 600 nm. The mannose-functionalized NLCs that have a mean diameter of 273 nm and are administered through the pulmonary route are likely to reach the deeper region of the lungs [14]. The PDI values for the INH-NLC and Man-INH-NLC formulations were estimated to be 0.289 ± 0.04 and 0.223 ± 0.02, respectively. A PDI value below 0.3 indicated the uniform dispersion of the particles (monodisperse) within the formulation. The INH-NLC formulation showed a positive zeta potential due to the positive amine group of octadecylamine. A significant decrease in zeta potential was observed for Man-INH-NLC after the process of mannosylation. This is because positively charged amine groups of octadecylamine reacted with the aldehyde group of D-mannose and caused shielding of the positive charge. This strongly suggests the successful mannosylation of the Man-INH-NLC formulation. Also, the positive charge of the mannosylated formulation enhances the cell internalization and intracellular accumulation of the drug within the infected cells, leading to an improvement in the anti-bacterial function of the drug [31,32]. Moreover, Man-INH-NLC was regarded as physically stable due to its steric stabilization and electrostatic repulsions between particles [33].

### 2.4. In Vitro Drug Release Analysis

**Figure 2:**
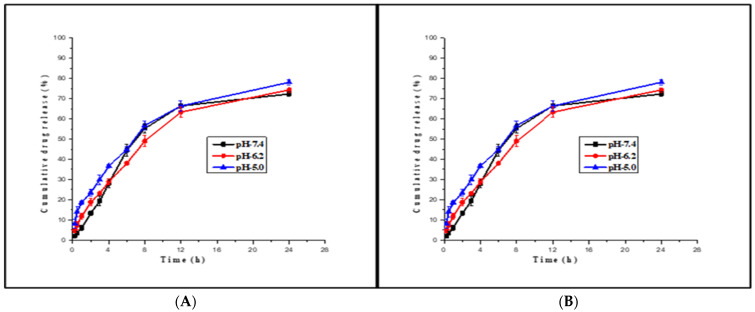
In vitro drug release studies of Man-INH-NLC (**A**) and INH-NLC (**B**) at pH 7.4 for lung fluid, pH 6.2 for phagosomes, and pH 5.0 for phagolysosomes (all the values are expressed as mean ± SD; *n* = 3).

The in vitro drug release from Man-INH-NLC was evaluated at different pH conditions of phosphate-buffered saline and compared with unfunctionalized INH-NLC, and the results are shown in Figure 2. It was observed that drug release from Man-INH-NLC (72.35 ± 1.09%) is slower compared to INH-NLC (78.11 ± 1.27%) in phosphate-buffered saline pH 7.4. Drug release was also analyzed at other pH levels (PBS pH 6.2 and 5.0) to simulate the behavior of the nanocarrier formulations in different parts of the lungs, and drug release was found to be slightly slower for Man-INH-NLC compared to INH-NLC. This could be a result of the extra outer coating of mannose around nanocarriers, which acts as a barrier, significantly slowing the release of the drug. The drug release from Man-INH-NLC had slight differences at all pH values and was comparable to the others, as shown in Figure 2A. A similar kind of effect can be seen in Figure 2B for the INH-NLC formulation. This led us to conclude that the drug release of both formulations was independent of the pH values and that both could be suitable drug carriers for pulmonary administration.

### 2.5. In Vitro Release Kinetics

The behavior of drug release from the nanocarrier formulation was examined using numerous mathematical models. For INH-NLC and Man-INH-NLC, the plots of log time versus log percent drug release were shown to be linear, with the highest correlation coefficients being 0.9661 and 0.9717, respectively (Table 3). The %DR for both INH-NLC and Man-INH-NLC was determined according to the Korsmeyer–Peppas model, and it was found that the drug was released by a non-Fickian diffusion-controlled mechanism with *n* values of 0.8086 and 0.8788, respectively [34].

### 2.6. Transmission Electron Microscopy

**Figure 3:**
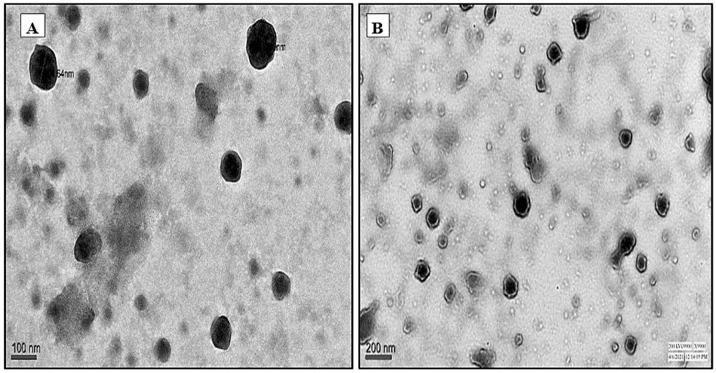
TEM micrographs of (**A**) INH-NLC and (**B**) Man-INH-NLC at 10× magnification.

TEM images of both INH-NLC and Man-INH-NLC showed spherical particles with uniform shapes and smooth surfaces. The coating around the INH-NLC nanocarriers can be seen in Figure 3B. The nanometric size of INH-NLC was increased after mannosylation. No evidence of particle agglomeration was found. As can be observed below, the morphologies of both the INH-NLC (Figure 3A) and Man-INH-NLC (Figure 3B) formulations are identical, showing that mannosylation does not affect particle shape and surface.

### 2.7. In Vitro Evaluation of Inhalation Behavior of Nebulized Mist

The Anderson cascade impactor was utilized to evaluate the in vitro inhalation properties of the mist formed from NLC dispersion produced by the nebulizer [35]. MMAD represents the value below which 50% of the particles are present in the respirable range. As per WHO guidelines, MMAD should be below 5.5 µm. MMAD of 2.3 ± 1.1 µm showed that 50% of the particles of the nebulized mist of the Man-INH-NLC dispersion were below the size of 2.3 ± 1.1 µm. GSD refers to the geographic mean of all aerosolized particles, which should be below 2.3 µm. A GSD value of 1.9 ± 0.2 µm showed that the geographic mean of all aerosolized particles was 1.9 ± 0.2 µm. The output efficiency represents the efficiency of the nebulizer in producing the mist of the nanoformulation, and it was found to be 95.24 ± 4.56%. Respirable fractions represent the inhalation characteristics of the formulation and provide the concentration of the formulation that reaches the lungs. The respirable fraction was found to be 87.33 ± 6.09% for the nebulizer. This study proved that the air jet nebulizer has high efficiency, provides good inhalation characteristics, and is suitable for the passive targeting of the Man-INH-NLC formulation. These values indicated the efficient delivery of nanocarrier-encapsulated drugs into deeper pulmonary regions [36].

### 2.8. In Vivo Pharmacokinetic Analysis

**Figure 4:**
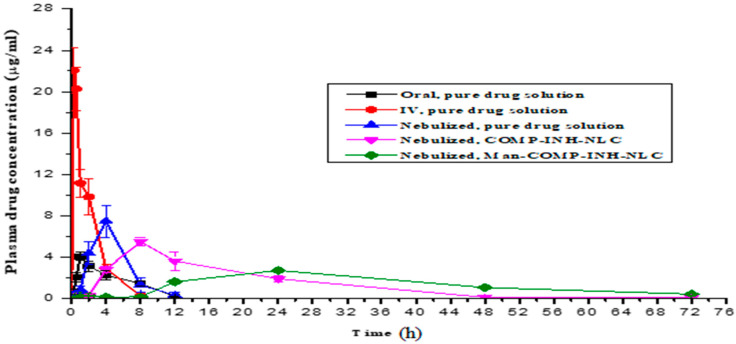
Plasma drug profile of isoniazid after single nebulization of Man-INH-NLC, INH-NLC, and parent drug. Values mean ± S.D.; *n* = 6 at each time point.

Pharmacokinetic parameters were analyzed for a comparative bioavailability assessment of the developed formulation. The results stated that, after a single nebulization, a very small amount of the drug was detected in the plasma for 4 h in the case of Man-INH-NLC formulation and 2 h in the case of the INH-NLC formulation, indicating its slow initial absorption (Figure 4). The delayed appearance of the drug in the blood was more notable for the Man-INH-NLC formulation in comparison to the INH-NLC formulation. The reason behind this is the effective coating of mannose and their affinity for mannose receptors, which increase the drug residence time in the lungs. The Man-INH-NLC and INH-NLC formulations remained in the therapeutic range for 48 h and 24 h, respectively. The longer stay of Man-INH-NLC is due to the encapsulation of the hydrophilic drug into a lipophilic matrix, and the additional mannose coating over it represents the greater sustained release effect of the drug compared to INH-NLC. In contrast, a relatively shorter stay of the parent drug in the lungs was observed after nebulization. The drug started to appear in plasma after one hour of pulmonary administration due to its highly hydrophilic nature and the non-availability of lipid encapsulation. The drug was not detected in the plasma as it was quickly eliminated from the blood after 12 h of nebulization due to very short t_1/2_.

The pharmacokinetic parameters of isoniazid are given in Table 4. C_max_ represents the maximum drug concentration that usually reaches the plasma after its administration, whereas *T_max_* is the time taken to achieve C*_max_* within the same. Man-INH-NLC gives rise to a shorter peak plasma concentration (C*_max_*) and takes a longer time to achieve it (T*_ma_*_x_). Among the pulmonary administered formulations, the highest C*_max_* (*p* < 0.05) was observed for the pure drug solution, followed by the INH-NLC formulation and, lastly, the Man-INH-NLC formulation, which resulted in the sustained release of the formulation due to its lipophilic nature. Additionally, the maximum C*_max_* (*p* < 0.05) was found for the intravenously administered pure drug solution, followed by the orally administered pure drug solution, and the former gets quickly eliminated from the body. K_el_ represents the rate at which a drug is removed from the human system. A low elimination rate value (K_el_) represented the extended t_1/2_ of any formulation. Man-INH-NLC has a slower elimination rate compared to INH-NLC. Mean residence time (MRT) indicated the average time taken by a drug to reside in the body. The MRT of the INH-NLC and Man-INH-NLC formulations experienced significant three-fold and seven-fold increases, respectively, when compared with the nebulized pure drug solution and four-fold and eleven-fold when compared to the orally administered parent drug solution. The highest AUC_0–∞_ value was found for the Man-INH-NLC formulation, followed by the INH-NLC, which affected both relative and absolute bioavailability. Relative bioavailability (compared to oral) after nebulization increased four- and five-fold for INH-NLC and Man-INH-NLC, respectively. Absolute bioavailability (compared to i.v.) also increased by nearly two- and three-fold for INH-NLC and Man-INH-NLC, respectively.

In vivo pharmacokinetic parameters bear important therapeutic implications and proved improved drug delivery with an enhanced residence time and improved pharmacokinetic profile with respect to the Man-INH-NLC formulation in the lungs as well as in blood compared to the unfunctionalized INH-NLC formulation and the pure drug solutions administered via oral and intravenous routes. This provided a better way to increase the therapeutic effect of isoniazid in the management of tuberculosis treatment [37].

### 2.9. Histological Evaluation

**Figure 5:**
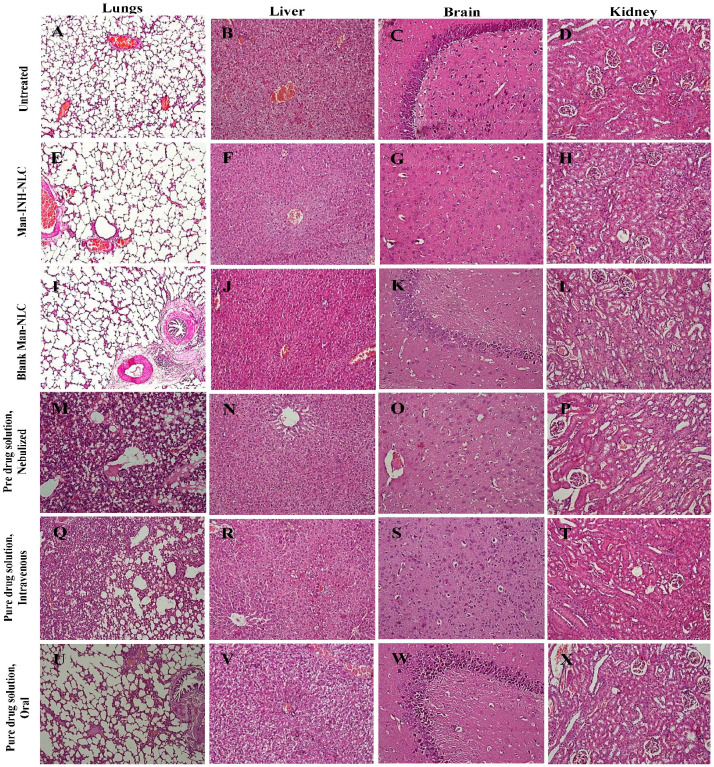
Histology of different organs (i.e., lung, liver, brain, and kidney) in each group of animals (guinea pig; *n* = 6) receiving different treatments viz. Untreated (**A**–**D**), Man-INH-NLC, Nebulized (**E**–**H**), Blank Man-NLC, Nebulized (**I**–**L**), Pure drug solution, Nebulized (**M**–**P**), Pure drug solution, Intravenous (**Q**–**T**), Pure drug solution, Oral (**U**–**X**). The generation of the images shown was facilitated by using a light microscope at 10× magnification.

Histological analysis was carried out at high doses of isoniazid (40 mg/kg/day) and administered in different formulations via a different route of administration. The evaluation was carried out for any kind of allergic reaction and toxicity to the lungs and other body organs after repeated administration for a long time. Figure 5A–D show the untreated (control) lung, liver, brain, and kidney tissues of the experimental animals, respectively. After 4 weeks of the daily nebulization of the Man-INH-NLC and blank Man-NLC formulations, some minor changes were observed in the alveolar area of the lungs, which were not toxic when compared to the untreated control. The interalveolar spaces and interalveolar septum of the lungs were found to be normal for both formulations after nebulization (Figure 5E,I). No kind of inflammation or degenerative changes were observed in the liver (Figure 5F,J) and brain (Figure 5G,K), resulting in its safe use. After the nebulization of the pure drug solutions, the lungs of the treated animals showed edema in alveolar spaces (Figure 5M). Mild changes in hepatocytes and low-grade central congestion were observed in the liver of the animals compared to the group receiving Man-INH-NLC (Figure 5N). No kind of histological changes were found in any part of the brain among the treated animals (Figure 5O). Animals receiving a pure drug solution via nebulization showed higher inflammation in their lungs than those treated with pure drug solutions administered intravenously (Figure 5Q). Metabolic changes and an increased concentration of glial cells resulted in increased inflammation being observable in the liver and brain, respectively. (Figure 5R,S). In the case of the orally administered pure drug solution, thickening of the interalveolar septum and vascular degeneration to a small extent were found in the lungs and liver, respectively (Figure 5U,V), whereas a degenerated nucleus was found in the cerebrum of the brain of the treated animals (Figure 5W). No kind of changes or signs of toxicity were observed in the kidneys for all of the formulations administered via a different route of administration. Glomerular filtration and glomerular tubule appeared normal. The solution responsible for causing the most toxicity to the liver and brain was found to be the orally administered pure drug solution, while the nebulized pure drug solution facilitated large toxicity in the lungs in contrast to other organs. It was concluded that treatment with a high dose of blank mannosylated nanocarrier formulation (Man-COMP-NLC) and a drug-loaded mannosylated nanocarrier formulation (Man-INH-NLC) via nebulization appeared to be well tolerated by the animals, having been found safe during the entire period of study. No toxicity was observed for the blank Man-NLC and Man-INH-NLC formulations due to the encapsulation of the drug in biocompatible/physiological lipids and the provision of a sustained release effect for a large duration of time.

### 2.10. Hepatotoxic and Nephrotoxic Evaluation

The parameters for hepato- and nephrotoxicity in serum were evaluated with respect to normal functioning of the liver and kidney, respectively. ALT/ALP/AST were used as indicators of liver function tests, and urea/creatinine/bilirubin were used as markers of kidney function tests. An increase in these parameters beyond normal values indicated the presence of toxicity or an allergic reaction in the respective organs. An evaluation of hepatotoxicity and nephrotoxicity was carried out for Man-INH-NLC, blank Man-NLC, and the pure drug solutions administered via different routes of administration, and the results are shown in Table 5. The results indicated a remarkable increase (*p* < 0.05) in serum ALT, ALP, and AST levels upon the administration of high doses of the pure drug solutions administered via oral, intravenous, and pulmonary routes in comparison with the untreated control group. Significant (*p* < 0.05) increases in serum ALT (38.64, 103.77, 97.06 IU/L), ALP (153.51, 379.99, 371.66 IU/L), and AST (34.71, 96.52, 103.68 IU/L) activities were found upon the administration of the pure drug solutions administered via the oral, intravenous, and pulmonary routes, respectively, indicating that a pure drug solution of isoniazid induces damage to hepatic cells possibly because the free drug was exposed to liver cells without any shielding effect to non-target cells, thereby leading to more toxic action. The administration of blank Man-NLC and Man-INH-NLC resulted in a slight increase of ALT, ALP, and AST to 39.78, 193.64, and 49.13 IU/L, respectively, which was found to be within the normal limits compared to the untreated control group because of the encapsulation of the drug in the biocompatible lipids and its sustained drug release effect from the lipid matrix. Furthermore, both blank and isoniazid-encapsulated Man-INH-NLC administered via the pulmonary route led to a slight increase in urea, bilirubin, and creatinine levels within normal ranges in serum compared to the untreated control. This may be due to the fact that the drug is directly targeted to the lungs, meaning that the drug’s access to the kidney was minimal. A moderate increase in the same parameters was found in the case of pure drug solutions administered via the oral, intravenous, and pulmonary routes, and this increase was found to be within the normal ranges. These results indicate that both the pure isoniazid drug solution and the drug encapsulated in the lipid nanocarrier formulation had no harmful effect on the kidney and failed to induce any toxic effects in kidney cells. These results are in close agreement with the results obtained in the histological studies and point towards the safety of administering Man-INH-NLC through the pulmonary route using a nebulizer.

## 3. Materials and Methods

### 3.1. Material and Components

Compritol 888 was procured as a gift sample from Gattefosse Pvt Ltd., Saint-Priest, France. Other chemicals, namely, isoniazid, octadecylamine, linoleic acid, tween 80, and poloxamer 407 were purchased from Merck, Burlington, MA, USA. All the chemicals and analytical reagent (AR) were of analytical grade. Double distilled water was used for all experiments.

### 3.2. Method of Preparation of NLC

The hot homogenization–ultrasonication technique was used to prepare the INH-NLC formulation. The concentrations of all of the lipids and drugs were optimized in our previous studies and used accordingly [38]. Briefly, all of the lipids (compritol-50.67 *w*/*w*, octadecyl amine-26.94 *w*/*w*, and linoleic acid-22.38 *w*/*w*), together with tween 80 (0.1 g), were heated to 80 °C under continuous stirring. The aqueous phase was constituted separately using poloxamer 407 (1%) in double-distilled water and heated at an identical temperature. This aqueous solution (1 mL) was used to dissolve the drug (INH- 239.82 mg), and this lipid melt was subsequently subjected to homogenization at 18,000 rpm (Heidolph, Schwabach, Germany) for 5 min to make w/o emulsion. A constant temperature was maintained throughout the entire procedure. The dropwise addition of a surfactant solution (aqueous phase) was carried out to obtain w/o/w emulsion and subjected to sonication by employing a probe sonicator (Q55, Sonica Sonicators, New York, NY, USA) at an amplitude of 80% for 5 min. This INH-NLC dispersion was subjected to cooling at room temperature to facilitate nanosuspension.

### 3.3. Mannosylation of Nanostructured Lipid Carriers

The mannose functionalization of INH-NLC was accomplished using a previously reported method with some modifications [17,39]. Firstly, a 50 Mm D-(+)-mannose solution was prepared in acetate buffer pH 4.0 and added to the INH-NLC dispersion till the concentration of octadecylamine remained at 0.02% *w*/*v* in the final INH-NLC dispersion. Secondly, this dispersion was subjected to continuous and gentle stirring for 48 h to obtain the completion of the reaction at room temperature. This dispersion was subjected to 70 °C temperature under vacuum to obtain a concentrated formulation. The removal of free mannose was achieved by exhibiting the Man-INH-NLC under extensive dialysis with double-distilled water for 45 min using an activated dialysis bag (molecular weight cut-off 12–14 k Da, Hi-Media, Mumbi, India). The resulting nanocarriers were freshly reconstituted in normal saline to facilitate nanosuspension [6]. The blank NLC formulation was formulated similarly, without the addition of INH.

### 3.4. Fourier-Transform Infrared Spectroscopy

FTIR spectroscopy was carried out to confirm the mannose coating on the Man-INH-NLC surface by analyzing the formation of Schiff’s base. FTIR analysis of INH-NLC and Man-INH-NLC was performed via the KBr pellet method using an FTIR spectrophotometer (Spectrum BX, Perkin Almer, New York, NY, USA). Analysis was carried out in a frequency range ranging from 4500 cm^−1^ to 350 cm^−1^ at 4 cm^−1^ resolutions with a sample/KBr ratio of 1:10 [40].

### 3.5. Encapsulation Efficiency and Drug Loading Analysis

A UV spectrophotometer (UV-1800, Shimadzu, Tokyo, Japan) was used to evaluate the %EE and %DL of both INH-NLC and Man-INH-NLC at λ_max_ 262 nm. Centrifugation of NLC suspension was carried out at 18,000 rpm for 45 min at 4 °C to obtain the NLC pellet. The NLC pellet was separated, and the below aqueous phase was subjected to a suitable solvent extraction method to recover the drug. The obtained drug samples were sufficiently diluted with distilled water, filtered through 45 µm filter paper, and analyzed for drug content [41].

(1) $$Entrapment\;efficiency\;\left(\%\right)=\frac{Wi-Wf}{Wi}\times 100$$

(2) $$Drug\;Loading\;\left(\%\right)=\frac{Wi-Wf}{Wi-Wf+Wt}\times 100$$

where *W_i_* = weight of drug added during NLC formation; *W_f_* = weight of unentrapped drug; *W_t_* = total weight of all lipids and drugs added to the NLC formulation [42].

### 3.6. Average Particle Size, Polydispersity Index, and Zeta Potential Analysis

The particle sizes of both the INH-NLC and Man-INH-NLC formulations were analyzed using a Zeta sizer (Nano ZS^®^, Malvern, UK) at 25 °C with backscattered light at 90°. This gives the average particle size as a z-average, also known as the average hydrodynamic diameter of a nanoformulation [43]. PDI represents the homogeneity of the dispersion, and its value ranges from 0 to 1. Values approaching 0 indicate relatively homogenous dispersion, while values larger than 0.5 indicate heterogeneous dispersion [44]. The zeta potential (ZP) indicates particle surface charge, and the Helmholtz–Smoluchowski equation was used to calculate particle electrophoretic mobility and convert it into zeta potential. Multiple scattering effects were prevented by making suitable dilutions with deionized water [45].

### 3.7. In Vitro Drug Release Analysis

The release of INH from both the INH-NLC and Man-INH-NLC formulations was investigated via the dialysis bag approach using an activated dialysis tube in phosphate-buffered saline (PBS) pH 7.4 for lung fluid, pH 6.2 for nasal fluid and phagosomes, and pH 5 for phagolysosomes to simulate the lung environment after pulmonary administration. The NLC dispersion was equivalent to 5 mg of INH and put into a double-folding dialysis bag that was carefully sealed on both sides and immersed in beakers carrying 250 mL of dissolution media. The stirring rate was set to 200 rpm, and the temperature was kept at 37 ± 0.5 °C. A parafilm was used to cover all the beakers to prevent solvent loss. The samples (5 mL) were withdrawn at different time points (0.25, 0.5, 1, 2, 3, 4, 6, 8, 12, and 24 h), and the same amount was substituted with fresh dissolution media after each withdrawal to maintain the sink condition. The amounts of INH released were analyzed spectrophotometrically [46].

### 3.8. In Vitro Drug Release Kinetics

Numerous release kinetic models (zero-order, first-order, Higuchi’s square root, and Korsmeyer–Peppas) were employed to investigate the behavior of drug release from the optimized formulation. The model with the best fit was determined based on which had the highest correlation coefficient (R^2^). The *n*-value indicated the behavior of drug release (*n* < 0.5 represents Fickian transport; *n* = 0.5 to 0.9 represents non-Fickian transport) in the Korsmeyer–Peppas model [34].

### 3.9. Transmission Electron Microscopy

TEM analyses of the INH-NLC and Man-INH-NLC formulations were performed to evaluate the particle sizes and internal morphologies of the formulations. A TEM instrument (Tecnai G2 F20 U-TWIN, Beijing, China) was used to capture the photomicrographs of the NLC formulation. A droplet of both INH-NLC and Man-INH-NLC dispersion was put on a copper grid (Boston Industries, Inc., Walpole, MA, USA). Filter paper was used to wipe off the excess droplets. After 60 s, uranyl acetate (2% *w*/*v*) was again put on the grid and subjected to drying. These samples were inserted and evaluated using TEM imaging analysis (TIA) software version 2 [47].

### 3.10. In Vitro Evaluation of Inhalation Behavior of Nebulized Mist

The air-jet nebulizer (OMRON Healthcare Inc., Kyoto, Japan) was used to nebulize the Man-INH-NLC dispersion, which was evaluated using an Andersen cascade impactor (AN-200, TISCH Environmental Inc., New York, NY, USA) with a throat-connecting tube. Cascade impactors may partially dry aqueous aerosol droplets due differences in temperature between the nebulizer outlet and the body of the impactor, resulting in the overestimation of the respirable fractions. So, the impactor was cooled for one hour before use to reduce the evaporation of aqueous aerosol droplets and increase the efficiency of the instrument [31]. A total of 5 mL of Man-INH-NLC dispersion was nebulized, and a vacuum was created in the system with an air stream of 28.3 L/min for 30 min. After complete nebulization, the formulation leftover in the nebulizer was measured. The NLCs deposited on the 0 to 6 stages of the instrument were washed out with 0.01 M hydrochloric acid (100 mL). The solution was diluted accordingly with the same. Also, 10 mL of the resultant solutions were centrifuged at 18,000 rpm for 45 min, and the aqueous phase was analyzed for drug content spectrophotometrically. The output efficiency and respirable fraction were established to express the spray and inhalation characteristics of the nebulizer (Equations (3) and (4)). MMAD is the diameter of the aerosol below which half of the particles are enclosed, and GSD is the geometric mean of all the aerosolized particles that were also evaluated [36].

(3) $$Output\;efficiency\;\left(\%OE\right)=\frac{INH\;loaded\;in\;the\;system-INH\;remained\;in\;the\;nebulizer}{INH\;loaded\;into\;the\;system}\times 100$$

(4) $$Respirable\;fraction\;\left(\%RF\right)=\frac{INH\;deposited\;on\;stages\;2\;to\;6}{INH\;loaded\;into\;the\;system}\times 100$$

### 3.11. Experimental Animal Model

#### 3.11.1. Animals

Dunkin Hartley male guinea pigs (400–500 g) were acquired from Chaudhary Charan Singh Haryana Agricultural University, Hisar, (India), and used for the present study. This animal protocol was approved with the letter minutes of IAEC/2020/10-18/01 by the Animal Ethics Committee of the institute, Guru Jambheshwar University of Science and Technology, Hisar. The animals were housed as per CPCSEA guidelines and fed on green vegetable-based diets, and water was supplied ad libitum. The animals were subjected to fasting for 24 h before study [48].

#### 3.11.2. Nebulization Conditions

An air jet nebulizer system (250 kPa pressure; airflow 5.5 L/min) was employed. Treatments were given to the animals according to the protocol. The sterilized isotonic solution was used to prepare a pure drug solution. The inhalable dose of NLC dispersion was calculated based on the percent respirable fraction. The formulation was exposed for 3–4 min per animal using a nebulizer with the help of an appropriate face mask. A nebulizer spacer was attached in between the nebulizer and face mask to keep the medicated mist within the spacer for a sufficiently long time and prevent environmental loss. The animals were exposed to NLC dispersion (2 mL) for 3–4 min/animal. Instead of the duration of administration, the size of the dose was determined by the volume of sterile isotonic solution used [49].

#### 3.11.3. In Vivo Pharmacokinetic Analysis

The guinea pigs were divided into six groups, each with six animals: Group 1, INH-NLC, administered via a nebulizer; Group 2, Man-INH-NLC, administered via a nebulizer; Group 3, blank Man-NLC, administered via a nebulizer; Group 4, pure drug solution, administered orally; Group 5, pure drug solution, administered intravenously (iv); Group 6: pure drug solution, administered via a nebulizer. An isoniazid dose equivalent to 10 mg/kg/day was used for this study. Upon drug administration, 0.5 mL of blood was withdrawn at appropriate time points (0.25, 0.5, 1, 2, 4, 8, 12-, 24-, 48-, and 72-h) following administration and collected in pre-heparinized microcentrifuge tubes. The whole blood was centrifuged at 5000 rpm for 10 min at 4 °C; the supernatant plasma was separated and stored at −20 °C until it was required for analysis. Drug content in the plasma was analyzed using ultra-sensitive high-performance liquid chromatography (U-HPLC) [37].

Pharmacokinetic parameters were estimated by using the plasma concentration–time isoniazid curve. Peak plasma concentration (C*_max_*) and the time taken to achieve C*_max_* (T*_max_*) were estimated directly from this curve. The elimination rate constant (K_el_) was estimated via regression analysis and half-life elimination (t_1/2_) was derived using 0.693/K_el_. The trapezoidal method was used to estimate the area under the concentration–time curve (AUC_0–t_). The terminal AUC_t–∞_ was obtained by dividing the last measurable plasma drug concentration by K_el_. The mean residence time (MRT) was estimated by dividing the area under the moment curve (AUMC) by the area under the curve (AUC). Both relative and absolute bioavailability were calculated using Equations (5) and (6) [50].

(5) $$Absolute\;bioavailability=\frac{AUC\;nebulizer}{AUC\;i.v.}\times \frac{Dose\;i.v.}{Dose\;nebulizer}$$

(6) $$Relative\;bioavailability=\frac{AUC\;nebulizer}{AUC\;oral}\times \frac{Dose\;oral}{Dose\;nebulizer}$$

### 3.12. Reverse-Phase High-Performance Liquid Chromatographic Condition

A previously reported simple, sensitive, and reliable RP-HPLC method was used to evaluate isoniazid (INH) content in blood plasma [51]. The HPLC system (Nexera X2, Shimadzu Corporation, Kyoto, Japan) consisted of a gradient pump, an online degasser, and an ultraviolet DAD detector, and an autosampler was used. Chromatographic separation was carried out using the reverse-phase column C-18 (250 mm × 4.6 mm; 3–5 μm particle size). Nicotinamide was used as an internal standard. The mobile phase consisted of water and methanol in an initial composition of 95:05 *v*/*v* at a flow rate of 1.5 mL/min for 12 min, followed by a composition change to 20:80 *v*/*v* for 23 min. At 23 min composition was again changed to the 95:05 *v*/*v* for 3 to 5 min before the next injection. The injection volume used was 100 μL, and the estimation was carried out at 262 nm for both INH and NA.

### 3.13. Method Validation and Preparation of Calibration Curve

Stock solutions (1.00 mg/mL) of both the drug and internal standard were prepared in water. Appropriate concentrations were prepared using different volumes of this stock solution and diluting them with water. Calibration standards of 0.5, 1.0, 2.5, 5.0, 10.0, and 20.0 μg/mL of INH were constructed by adding 45 μL of the standard and 5 μL of the internal standard solution to 150 μL of a blank plasma. Three quality control (QC) samples of isoniazid were prepared using blank plasma: low (2.0 μg/mL), middle (5.0 μg/mL), and high (12.0 μg/mL). The lower limit of quantification (LLOQ) and limit of detection (LOD) were also calculated. LLOQ is the lowest concentration on the calibration curve (estimated with an accuracy of above 80% with precision below 20%). LOQ is defined as a signal-to-noise ratio (S/N) of 3:1.

### 3.14. Preparation of Plasma Samples

The plasma samples were prepared by adding 195 μL of plasma and 5 μL of internal standard solution and subsequently thawed either fresh or at room temperature. Afterwards, 40 μL of acetonitrile, 160 μL of zinc sulfate (10% in water), and 5 μL of ammonia (25%) were added separately and vortexed for 1 min after each addition. The samples were centrifuged at 14,000 rpm for 15 min at 5 °C. The clear supernatants were stored at 5 °C, and each sample was removed from the cooling system 5 min before injection. The samples were then injected (100 μL) into the HPLC system.

### 3.15. Histological Evaluation

For the histological studies, the Man-INH-NLC formulation was evaluated for any kind of allergic reaction and toxicity to lung tissue. For this, the animals were divided into six groups (*n* = 6), and a high dose of isoniazid (40 mg/kg/day) was administered to each animal. The details of the groups were as follows: Group 1—high dose of pure drug solution, oral; Group 2—high dose of pure drug solution, i.v.; Group 3—high dose of pure drug solution, nebulizer; Group 4—high dose of Man-INH-NLC, nebulizer; Group 5—blank Man-NLC, nebulizer; Group 6—untreated control. The treatments were administered for 4 weeks once daily to simulate the long-term treatment of isoniazid. The next day of the last dosage administration, the animals were euthanized after an overdose of pentobarbitone sodium injection. The lungs, liver, kidney, and brain were recovered and preserved in a 10% buffered formalin solution. Sections of 5 μm were cut using a microtome and embedded in paraffin. The tissues were stained using hematoxylin-eosin (H&E), and the sections were investigated for granulomas, gross lesions or inflammation, and the degree of necrosis was determined by a certified pathologist who was completely oblivious to the treatment group [28,52].

### 3.16. Hepatotoxic and Nephrotoxic Evaluation

Hepatotoxicity and nephrotoxicity were evaluated simultaneously with histological evaluation. After 4 weeks of treatment, 1 mL of blood was withdrawn from each animal and subjected to a liver function test and kidney function test to evaluate any toxic effects and allergic reactions to the liver and kidney tissues, respectively. The blood samples were immediately processed by a certified pathologist to analyze total urea; bilirubin; creatinine for hepatotoxic evaluation; and alanine aminotransferase (ALT), alkaline phosphatase (ALP), and aspartate transaminase (AST) for evaluating nephrotoxicity in the animal groups. The normal range of these parameters was evaluated via the control group [50].

### 3.17. Statistical Analysis

Statistical data were analyzed using Prism 8.4.0^®^ software. Analysis of variance was carried out via one-way ANOVA–post-Dunnett to differentiate two or more experimental groups. The data were considered statistically significant at *p* < 0.05. All values are displayed as the mean and standard deviation (mean ± SD where *n* = 3 or 6).

## 4. Conclusions

The isoniazid-loaded NLC formulation was formulated and successfully functionalized with D-mannose. The Man-INH-NLC formulation was found to have an encapsulation efficiency of 79.71 ± 1.65% with an average particle size of 273.4 ± 8.24 nm. In vitro release studies showed the non-Fickian pH-independent sustained release of drugs from the nanocarrier formulation. The majority of the nebulized nanoparticles were found in the respirable range for delivering the encapsulated drug deep into the lungs. In vivo studies revealed that the Man-INH-NLC formulation increased the mean residence time of the drug into the lungs compared to the non-functionalized INH-NLC formulation. The other pharmacokinetic parameters (relative bioavailability, half-life, AUC, T*_max_*, C*_max_*) of Man-INH-NLC were also improved after encapsulating isoniazid into lipid nanocarriers when compared with pure drug solution administered via different routes of administration. Furthermore, no toxicity was observed for both blank Man-NLC and the drug-loaded Man-INH-NLC formulations after repeated nebulization during the entire study period, as revealed following biochemical hepatotoxicity evaluation and histopathological evaluation. From the results, it can be concluded that the Man-INH-NLC formulation can safely improve the dosage regimen and substitute the conventional formulations for better therapeutic efficacy in the management of tuberculosis.

### Future Prospects

The pulmonary route is a growing alternative to the oral or injectable administration of anti-tubercular drugs as it provides targeted drug delivery, reduces systemic side effects, and improves patient compliance. Tuberculosis treatment often involves a combination of multiple drugs to combat drug resistance and improve treatment outcomes. So, combination drug therapy can be applied to this research work to deliver therapeutics directly to the lungs to achieve the synergistic effect and enhance treatment effectiveness. Personalized medicine approaches and optimized drug delivery strategies can also be applied to allow for tailored treatment regimens.

## Figures and Tables

**Table 1:** Investigation of INH-NLC and Man-INH-NLC formulations for %EE and %DL.

S. No.	Formulation	Encapsulation Efficiency (%EE)	Drug Loading (%DL)
1.	INH-NLC	82.09 ± 3.60 **	18.39 ± 0.81 **
2.	Man-INH-NLC	79.71 ± 1.65 **	17.86 ± 0.37 **

** *p* > 0.05. (One-way ANOVA, post-Dunnet test). Values are expressed as mean ± SD; *n* = 3.

**Table 2:** Characterization of INH-NLC and Man-INH-NLC formulations in terms of average PS, PDI, and ZP.

S. No.	Formulation	Particle Size Analysis (nm)	The Polydispersity Index (PDI)	Zeta Potential (mV)
1.	INH-NLC	247.6 ± 4.05	0.289 ± 0.04	+42.48 ± 1.86
2.	Man-INH-NLC	273.4 ± 8.24	0.223 ± 0.02	+24.18 ± 2.26

Values are expressed as mean ± SD; *n* = 3.

**Table 3:** Correlation coefficient (r^2^) of various kinetic drug release models for different formulations.

S. No.	Formulation	Zero-Order	First-Order	Higuchi’s Square Root Model	Korsmeyer–Peppas Model
1	INH-NLC	0.739	0.739	0.9019	0.9661
2	Man-INH-NLC	0.7881	0.7881	0.9298	0.9717

**Table 4:** Salient pharmacokinetic parameters of isoniazid following a single nebulization of Man-INH-NLC compared with a free drug.

Formulations	C*_max_*, mg/L	T*_max_*, Hour	K_el_	t_1/2_, Hour	MRT, Hour	AUC_0–∞_ (mg.h/L)	Relative Bioavailability	Absolute Bioavailability
Pure drug solution, Oral	4.08 ± 0.42	1	0.16 ± 0.01	4.25 ± 0.34	4.87 ± 0.2	22.65 ± 2.55	1	0.49
Pure drug solution, IV	22.04 ± 2.21	0.25	0.66 ± 0.19	1.12 ± 0.3 **	1.88 ± 0.14	45.87 ± 3.97	ns	1
Pure drug solution, Nebulized	7.46 ± 1.55	4	0.093 ± 0.04 **	8.53 ± 3.33 **	7.43 ± 3.11 **	42.03 ± 9.70	1.86	0.92
INH-NLC, Nebulized	5.51 ± 0.4 **	8	0.027 ± 0.01	26.80 ± 6.39	18.81 ± 2.91	100.85 ± 3.83	4.45	2.10
Man-INH-NLC, Nebulized	2.72 ± 0.24 **	24	−0.0174 ± 0.001	40.06 ± 3.78	51.11 ± 2.46	118.61 ± 8.28	5.24	2.89

** *p* > 0.05. (One-way ANOVA, post-Dunnet with respect to the oral-free drug). Values are expressed as mean ± SD; *n* = 6; ns— not significant.

**Table 5:** Hepatotoxic and nephrotoxic evaluation in animals treated with a pure drug solution, blank Man-NLC, and Man-INH-NLC.

Formulation Code	Liver Function Test			Kidney Function Test		
	ALT (IU/L)	ALP (IU/L)	AST (IU/L)	Urea (mg/dL)	Bilirubin (mg/dL)	Creatinine (mg/dL)
Untreated control	38.64 ± 5.33	153.51 ± 10.29	34.71 ± 3.62	15.17 ± 1.36	0.39 ± 0.07	1.13 ± 0.09
The oral, pure drug solution	103.77 ± 7.21	379.99 ± 13.47	96.52 ± 10.72	25.63 ± 2.68	0.62 ± 0.16	1.65 ± 0.10
IV, the pure drug solution	97.058 ± 5.64	371.66 ± 14.75	103.68 ± 3.38	27.69 ± 3.36	0.68 ± 0.15	1.75 ± 0.13
The nebulized, pure drug solution	98.47 ± 8.61	359.25 ± 5.73	87.27 ± 7.39	23.71 ± 2.91	0.61 ± 0.17	2.01 ± 0.24
Nebulized, blank Man-NLC	53.64 ± 2.81 **	171.12 ± 10.75	42.77 ± 7.87	16.43 ± 1.13 **	0.48 ± 0.03 **	1.25 ± 0.12 **
Nebulized, Man-INH-NLC	39.78 ± 6.26	193.64 ± 5.81	49.13 ± 9.44 **	18.98 ± 0.67	0.43 ± 0.09 **	1.31 ± 0.12

Values are expressed as mean ± SD; *n* = 6. ** represents *p* > 0.05 (one-way ANOVA, post Dunnet) with respect to the untreated control. The normal ranges in guinea pigs—ALT: 10 to 90 IU/L; ALP: 80 to 350 IU/L; AST: 10 to 90; urea: 9 to 32 mg/dL; total bilirubin: 0.3 to 1.0 mg/dL; creatinine: 0.6 to 2.2 mg/dL.

## Data Availability

Data is contained within the article.
